# Different contributions of autophagy to retinal ganglion cell death in the diabetic and glaucomatous retinas

**DOI:** 10.1038/s41598-018-30165-7

**Published:** 2018-09-06

**Authors:** Hae-Young Lopilly Park, Jie Hyun Kim, Chan Kee Park

**Affiliations:** Department of Ophthalmology, Seoul St. Mary’s Hospital, College of Medicine, The Catholic University of Korea, Seoul, Korea

## Abstract

Diabetes mellitus and glaucoma are the two major causes of selective retinal ganglion cell (RGC) death. To determine the relationship between autophagy and RGC death, we compared autophagy and the related molecular pathways in diabetic and glaucomatous retinas and examined their effect on RGC survival. Biochemical analysis of microtubule-associated protein light chain 3 (LC3)-II and beclin-1 were observed. To determine the pathways involved in autophagy induction, adenosine monophosphate-activated protein kinase (AMPK) and the mechanistic target of rapamycin (mTOR) were also explored. Beclin-1 and the LC3B-II to LC3B-I ratio significantly elevated at 4 and 8 weeks after glaucoma induction; however, only a slight increase was apparent in the diabetic retina. Significant upregulation of phosphorylated AMPK and downregulation of phosphorylated mTOR was evident in the diabetic retina. After autophagy was inhibited with 3-methyladenine (3-MA), apoptosis of RGCs was significantly increased in the diabetic retinas. However, 3-MA inhibition of autophagy decreased the apoptosis of RGCs in glaucomatous retinas. Therefore, our results suggest that RGC death is differentially regulated by autophagy and that the pathways involved differ depending on the triggering injury.

## Introduction

Retinal ganglion cell (RGC) death occurs in a variety of ocular diseases. Diabetes mellitus and glaucoma are the two major causes of selective RGC death in the retina. These two diseases are also the most common causes of irreversible blindness worldwide. Therefore, understanding the mechanism of RGC death in these diseases will be important for saving patients’ vision. Glaucoma is a neurodegenerative disease characterized by elevated intraocular pressure (IOP). High IOP induces collapse and compression of the optic nerve, which leads to axonal transport disturbance of RGCs and subsequent growth factor deprivation, triggering RGC apoptosis^1–3^. Diabetic retinopathy results in microvascular changes, which are accompanied by RGC loss, reactive gliosis, and inner retinal thinning^4,5^. In the early stages of diabetes in the retina, selective RGC loss is observed without overt microvascular changes^6–9^. The role of defective energy control and metabolic status in RGC death in the diabetic retina are under extensive investigation.

Autophagy is regarded to have an important role in various organs^10,11^. Autophagy plays a housekeeping role which maintains the homeostasis of cells by eliminating damaged organelles and unwanted molecules and managing turnover of proteins^12^. However, activation of autophagy can be initiated by various stress injuries, such as starvation, ischemia, oxidative stress, low adenosine triphosphate (ATP) levels, growth factor deprivation, and accumulation of misfolded proteins^13–15^. In some circumstances, activation of autophagy can result cell death, which is called type II programmed cell death or autophagic cell death^10,16^. Studies investigating the role of autophagy have produced conflicting results to RGC death in the diabetic and glaucomatous retina^17^. However, whether autophagy promotes survival by restoring cell function or triggers autophagic cell death mechanism may be related to the initial injury. In a study with an animal model of glaucoma with chronic and mild elevated IOP, autophagy promoted cell death^18^. In this situation, the injury may have triggered growth factor deprivation in the RGCs. When growth factor deprivation occurs, the phosphoinositide 3-kinase (PI3K) pathway is downregulated, and B-cell lymphoma 2 (Bcl-2)/B-cell lymphoma-extra large (Bcl-xL) are decreased, which releases baclin-1 and induces beclin-1-related autophagy^19,20^. When optic nerve transection or crush is induced in RGC axons, autophagy plays a protective role^21,22^. With these injuries, intracellular calcium is thought to play a role in RGC death. However, autophagy can promote cell survival or cell death following ischemia or reperfusion injury in RGCs^23,24^. Similarly, autophagy in ischemic diabetic retinas has been reported to promote both cell survival and cell death. When the damage is the result of ischemia, retinal energy depletion and oxidative stress occur. Adenosine monophosphate-activated protein kinase (AMPK) functions as a metabolic sensor that signals energy deprivation, decreases mechanistic target of rapamycin (mTOR) activity, and initiates autophagy to restore energy^25,26^. To determine the role of autophagy in RGCs, we compared autophagy in diabetic and glaucomatous retinas, examined related molecular pathways, and determined the effects on RGC survival.

## Results

### Animal model confirmation and quantification of RGC loss

Glaucomatous eyes sustained elevation of IOP for the duration of the 8-week experiment. Baseline IOP was 16.5 ± 1.96 mmHg, and it increased gradually to 30.7 ± 2.08 mmHg after 1 week following cauterization. Elevated IOP maintained for 8 weeks (31.2 ± 2.16 mmHg) in cauterized eyes. The IOP of control eyes that underwent sham surgery was within the normal range of IOP during the course of experiment.

The weights and blood glucose levels of the diabetic rats were measured. At week 8 following streptozotocin (STZ) injection, the weight (321.7 ± 11.2 g) had decreased and the blood glucose levels (530.9 ± 14.3 mg/dL) were significantly higher in diabetic rats compared with control rats (442.3 ± 9.1 mg/dL and 132.7 ± 5.2 mg/dL, respectively).

**Figure 1:**
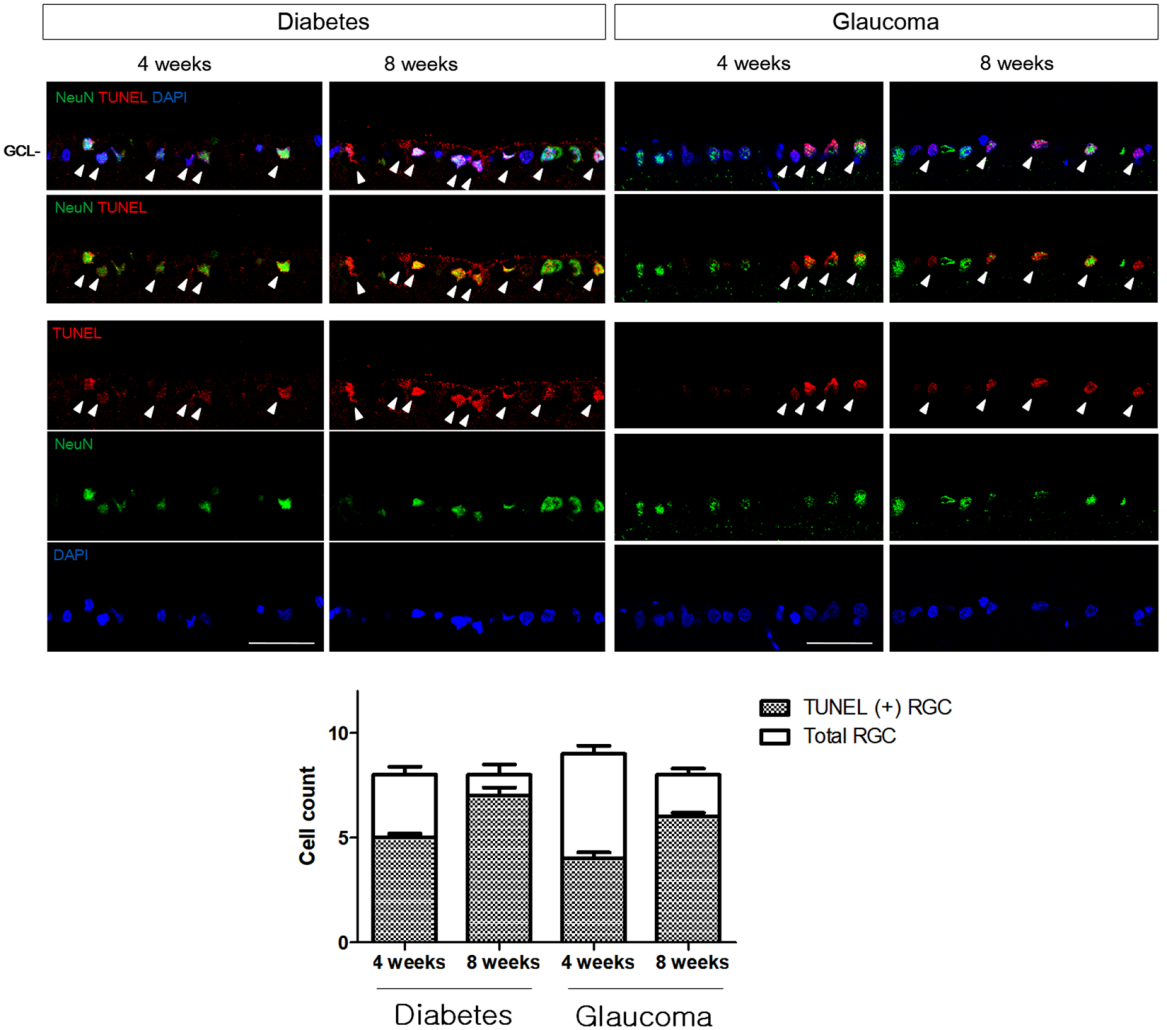
Terminal deoxynucleotidyl transferase dUTP nick-end labeling (TUNEL) staining of retinal ganglion cell (RGC) apoptosis. Double staining with NeuN identified RGCs in the ganglion cell layer (GCL) (white arrowheads). TUNEL-positive RGCs were found in the GCL in both diabetic and glaucomatous retinas, and the number of TUNEL-positive RGCs increased significantly in weeks 4 and 8 following induction of diabetes and glaucoma. Six eyes were used for each experimental period. Scale bars: 50 μm.

A significant increase in the number of apoptotic cells was identified in the ganglion cell layer (GCL) in weeks 4 and 8 following STZ injection and episcleral vein cauterization (Fig. 1). The number of terminal deoxynucleotidyl transferase dUTP nick-end labeling (TUNEL)-positive cells in the GCL was greater in the diabetic retinas than in the glaucomatous retinas in weeks 4, and 8. Following co-labeling with TUNEL and NeuN, apoptotic cells in the GCL were identified primarily as RGCs in both the diabetic and glaucomatous retinas.

### Expression of beclin-1 and microtubule-associated protein I light chain 3 β (LC3B-II)

**Figure 2:**
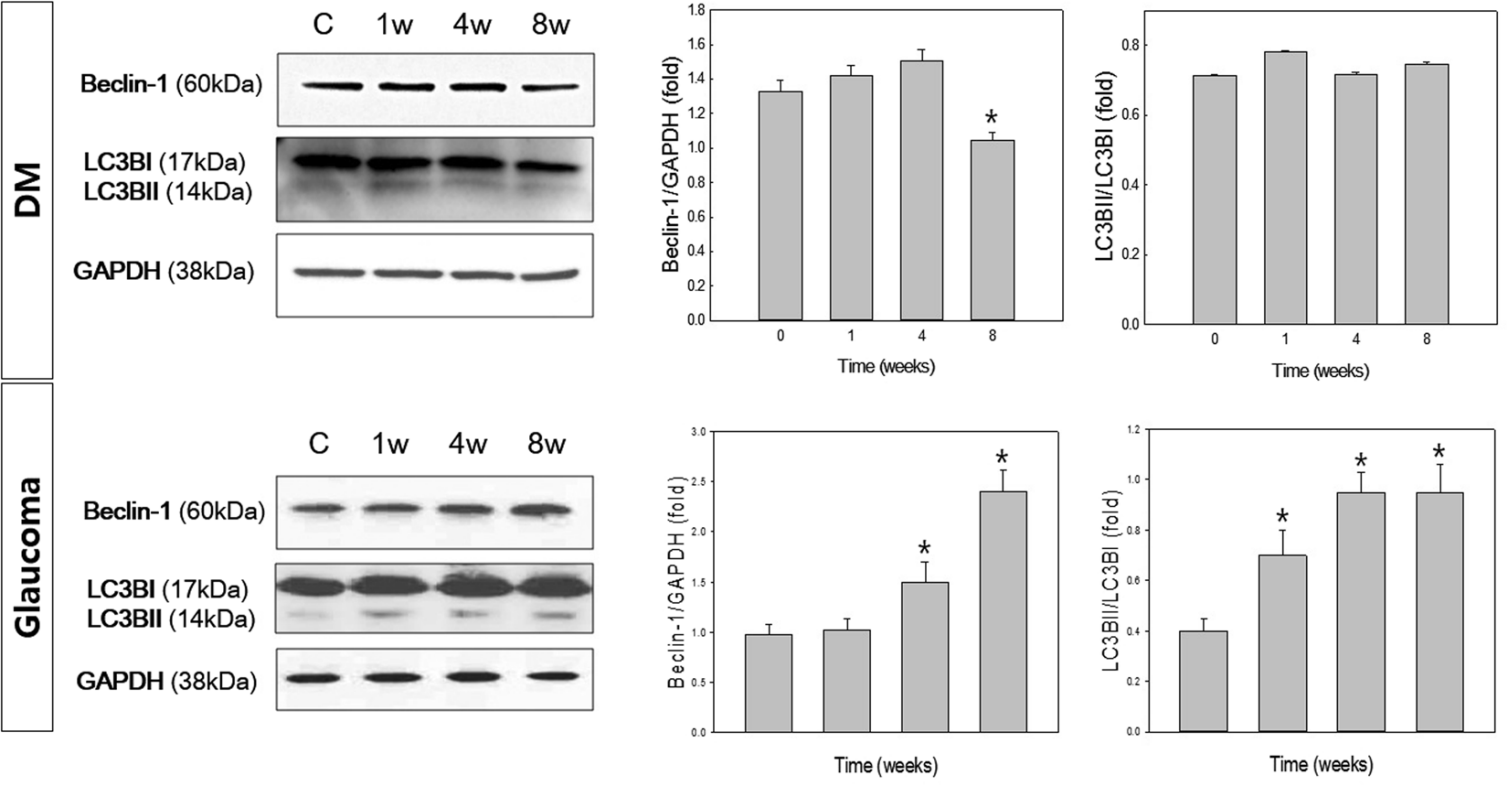
Analysis of beclin-1 and LC3B-II protein levels. GAPDH was used as an endogenous control. The level of beclin-1 relative to GAPDH and the LC3B-II-to-LC3B-I ratio were calculated at each time point, and both showed a significant increase at weeks 4 and 8 following induction of glaucoma. The level of beclin-1 and the LC3B-II-to-LC3B-I ratio also increased to a steady state in the diabetic retina, which did not show any significant changes over the course of the experiment, with the exception of a significant decrease in beclin-1 8 weeks after diabetes was induced. Six eyes were used for each experimental period. Bar represents the mean ± standard deviation (SD). Post hoc multiple comparison tests were used for statistical analysis. *p < 0.05 compared with the control. (See supplementary material).

We examined changes in beclin-1 and LC3B-II before and after induction of diabetes and glaucoma (Fig. 2). We found slight increase in Beclin-1 and the ratio of LC3B-II-to-LC3B-I at weeks 1, 4, and 8 following induction of diabetes compared with the controls. However, after 8 weeks, the level of beclin-1 decreased significantly, to a subnormal level, in the diabetic retina. The expression pattern of beclin-1 and LC3B-II differed in glaucomatous retinas. Beclin-1 and the ratio of LC3B-II-to-LC3B-I gradually increased in glaucomatous retinas. It was significantly elevated in weeks 4 and 8 following induction of glaucoma compared with the controls.

### Ultrastructural features of autophagy

**Figure 3:**
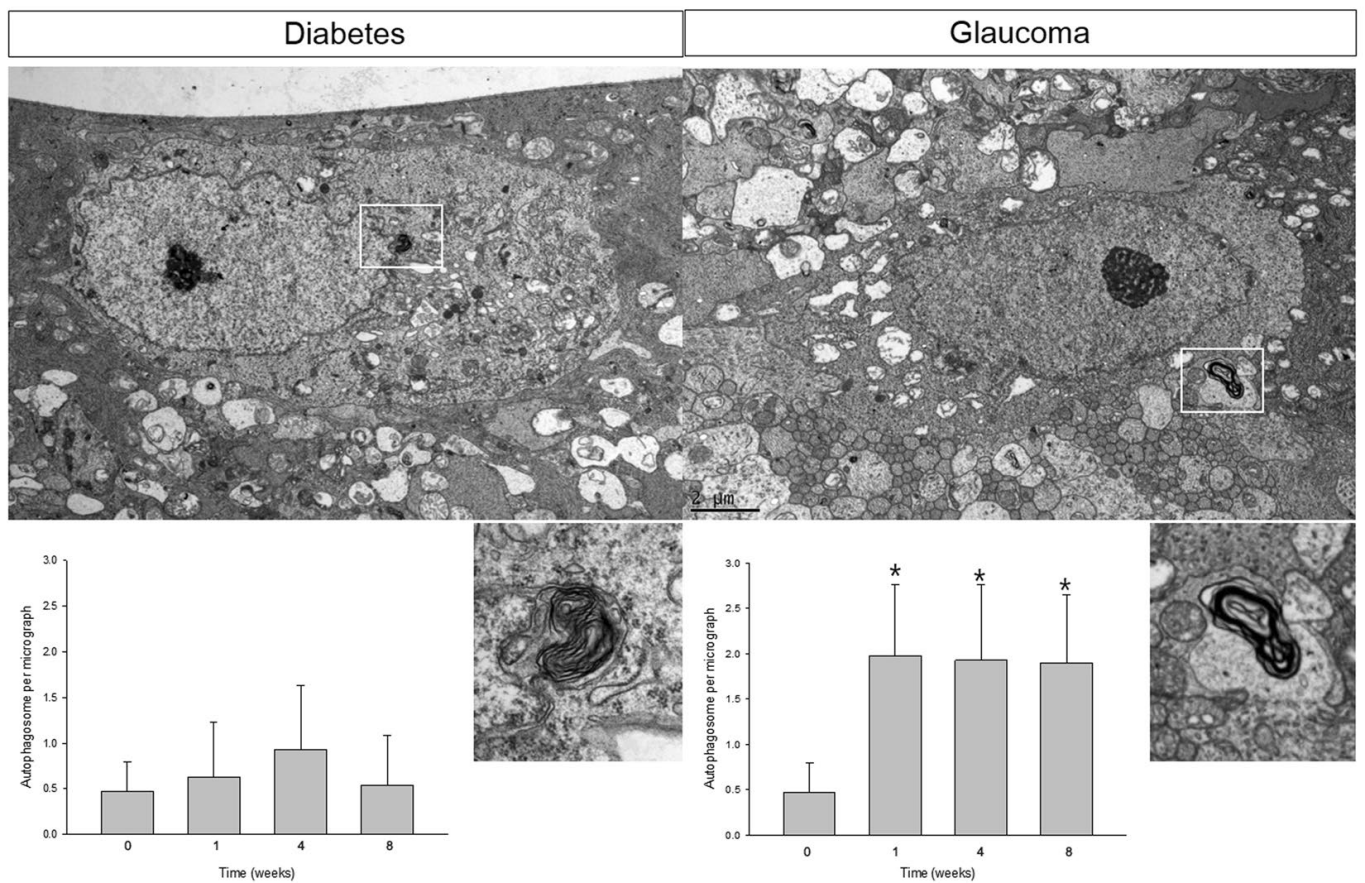
Transmission electron micrographs of retinal ganglion cells (RGCs) in the ganglion cell layer (GCL). Ultrastructural images show the presence of double- or multiple-membrane autophagic vesicles containing cell organelles (box) in the cytoplasm of RGCs in the GCL of diabetic and glaucomatous retinas. The number of autophagic vesicles per transmission electron microscopy (TEM) micrograph (50 μm^2^) in the diabetic and glaucomatous retinas is shown. A significant increase in the number of APs in glaucomatous retinas was apparent in weeks 1, 4, and 8. Six eyes were used for each experimental period. Bar represents the mean ± SD. Post hoc multiple comparison tests were used for statistical analysis. *p < 0.05 compared with the control. Scale bars: 2 μm.

To investigate the change in autophagic activity following induction of diabetes and glaucoma, we examined the presence of autophagosome (AP) using transmission electron microscopy (TEM). APs are double-membrane vacuoles that contain cytoplasmic structures, which represents the hallmark of autophagy^27,28^. We counted APs in each grid of the TEM up to 50 girds in total (50 micrographs, 2500 μm^2^ total) and found a significant increase in the number of APs compared with the controls following glaucoma induction (Fig. 3). The number of APs was 0.48/50 μm^2^ in the RGCs before induction of glaucoma. However, this increased and peaked to 1.98/50 μm^2^ at 1 week after glaucoma induction (Fig. 2B,D). This elevated expression of APs in the RGCs remained until 8 weeks after glaucoma induction, whereas these changes were not observed in the diabetic retinas.

### LC3 Distribution in Retinal Sections

**Figure 4:**
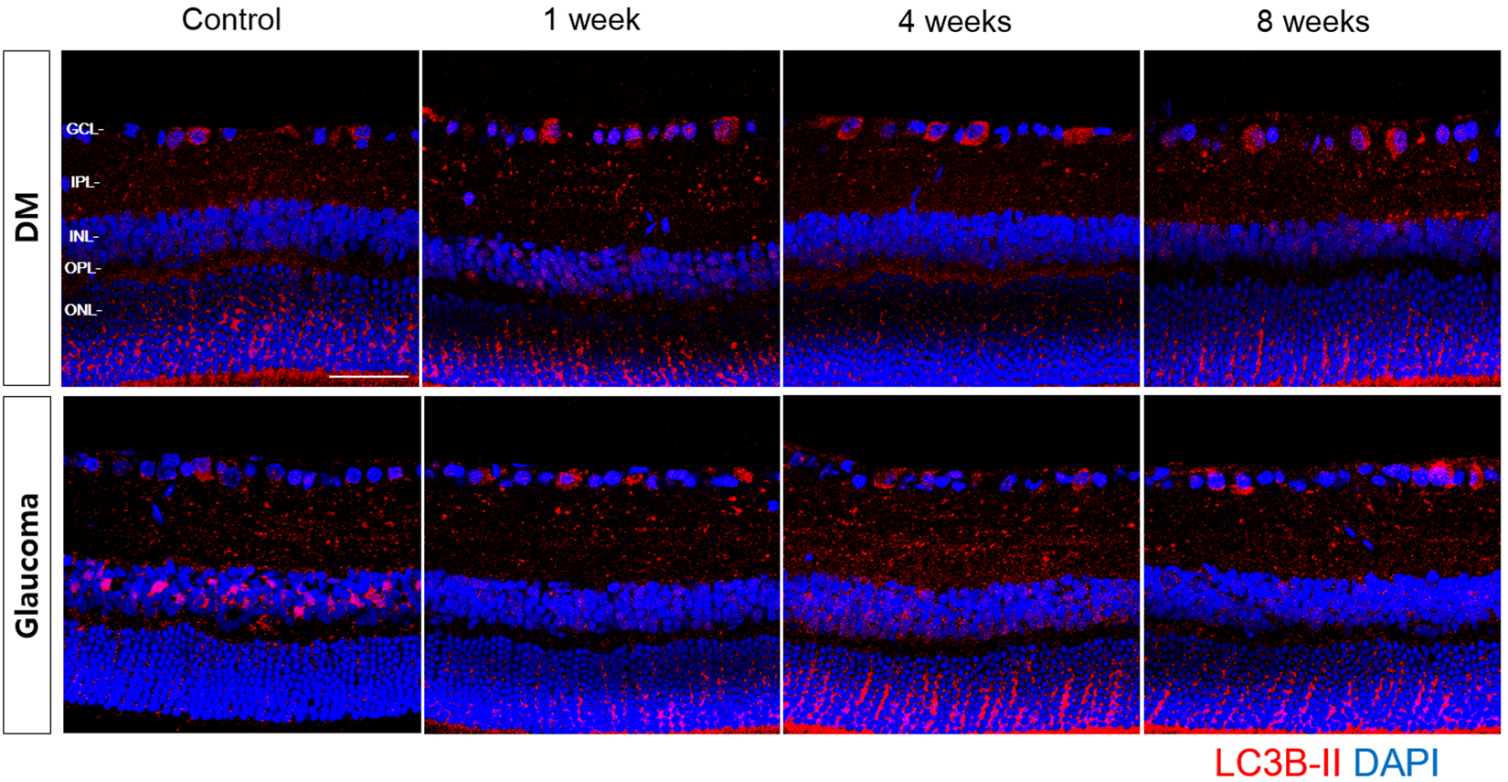
Time course of LC3B-II immunoreactivity revealed by confocal microscopy following induction of diabetes and glaucoma. LC3B-II immunoreactivity increased in the GCL and the inner plexiform layer (IPL) in both diabetes and glaucomatous retinas. INL, inner nuclear layer; OPL, outer plexiform layer; ONL, outer nuclear layer. Three eyes were used for each experimental period. Scale bars: 50 μm.

**Figure 5:**
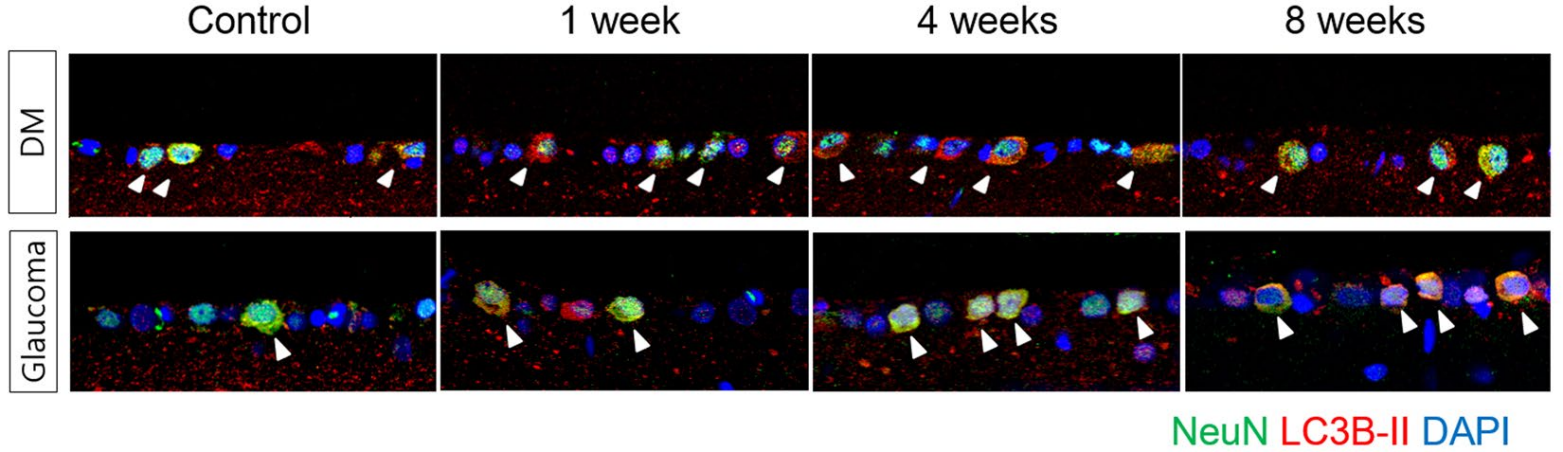
Confocal micrographs of LC3B-II and NeuN, a ganglion cell marker. In both diabetic and glaucomatous retinas, punctuate LC3B-II was located primarily in the cytoplasm of RGCs in the GCL, which increased following induction of diabetes and glaucoma. Three eyes were used for each experimental period. Scale bars: 50 μm.

We found LC3B-II expression throughout the RGCs in the GCL, although it was primarily identified in the inner plexiform layer (IPL) in the control retinas (Fig. 4). Following the induction of diabetes and glaucoma, LC3B-II expression significantly increased in the GCL. In the glaucomatous retinas, LC3B-II expression increased in both the GCL and the IPL, but it was more apparent in the IPL. Colocalization with NeuN indicated that increase in LC3B-II expression was in the cytoplasm of RGCs (Fig. 5). The expression of LC3B-II immunoreactivity increased following induction of diabetes and glaucoma (Fig. 5, white arrowheads).

### Expression of AMPK and mTOR

**Figure 6:**
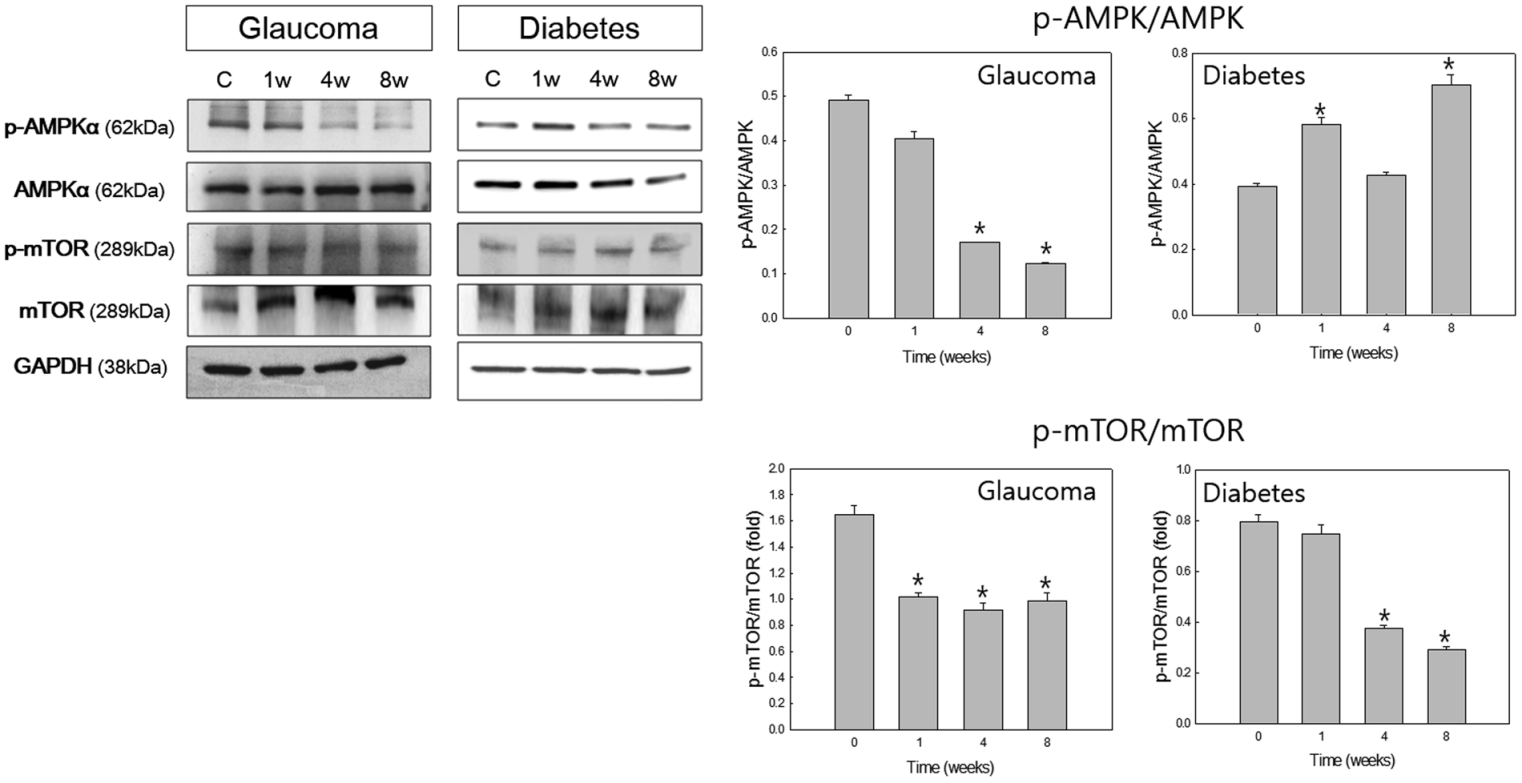
Western blot analysis of adenosine monophosphate-activated protein kinase (AMPK) and the mechanistic target of rapamycin (mTOR). The ratio of the phosphorylated forms relative to the total form was calculated. Phosphorylated AMPK (p-AMPK) was significantly decreased at weeks 4 and 8 following glaucoma induction. p-AMPK was significantly increased in weeks 1 and 8 after the induction of diabetes. Phosphorylated mTOR (p-mTOR) was significantly decreased in both diabetic and glaucomatous retinas. Six eyes were used for each experimental period. Bar represents the mean ± SD. Post hoc multiple comparison tests were used for statistical analysis. *p < 0.05 compared with the control. (See supplementary material).

To determine the involvement of the AMPK and mTOR pathways in the induction of autophagy, we assessed the change in the ratio of phosphorylated to nonphosphrylated forms of AMPK and mTOR (Fig. 6). Phosphorylated mTOR (p-mTOR) was significantly decreased in both diabetic and glaucomatous retinas compared with controls. However, phosphorylated AMPK (p-AMPK) was significantly decreased in glaucomatous retinas at weeks 4 and 8, whereas it was significantly increased in diabetic retinas at weeks 1 and 8 compared with controls.

### AMPK distribution in retinal sections

**Figure 7:**
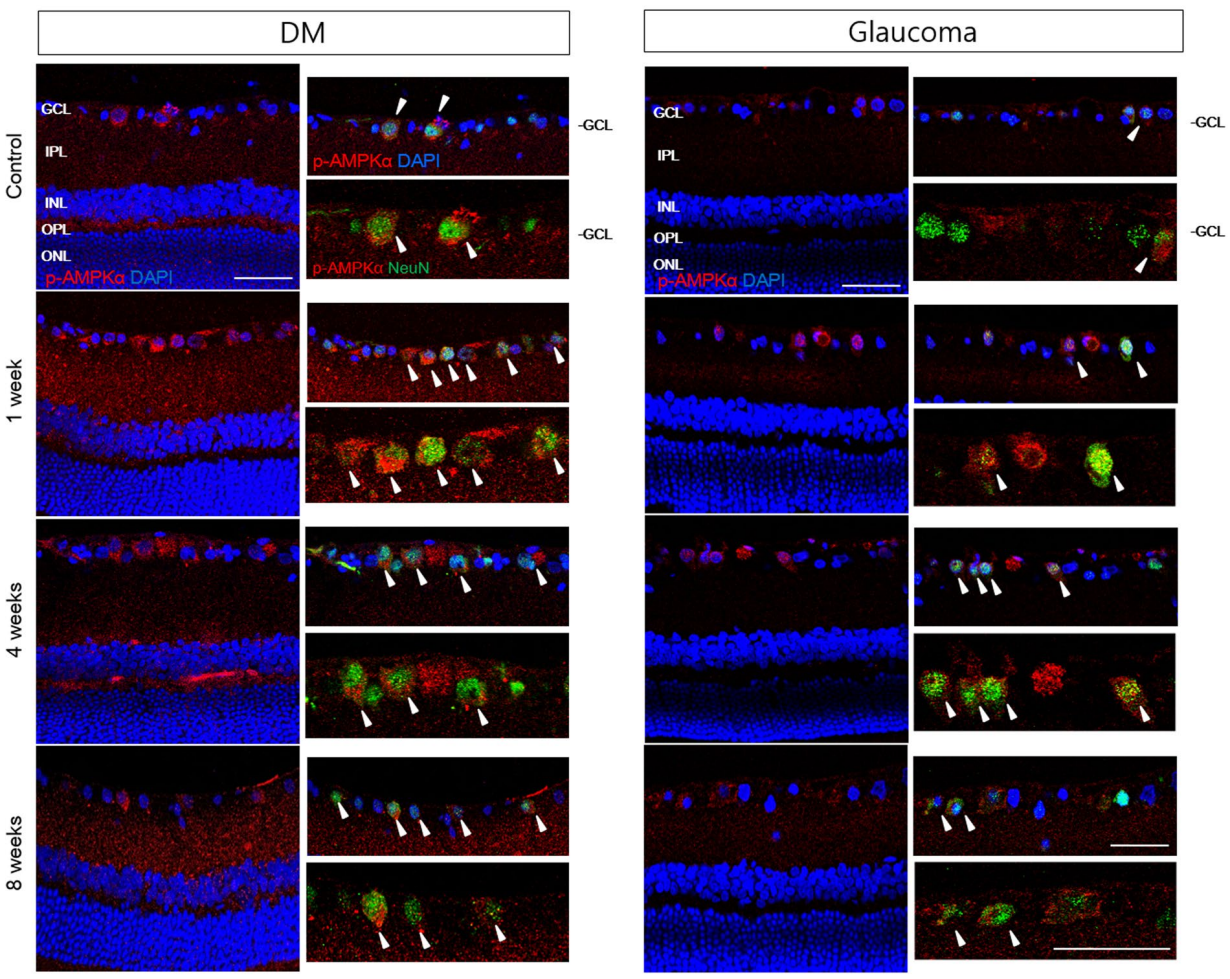
Confocal micrographs of labeled p-AMPK and NeuN. Immunoreactivity of p-AMPK was significantly upregulated in the diabetic retina compared with the glaucomatous retina throughout the course of the experiment. Colocalization with p-AMPK and NeuN significantly increased following induction of diabetes and glaucoma (white arrowheads). The increase in p-AMPK-positive RGCs was more significant in the diabetic retina. INL, inner nuclear layer; OPL, outer plexiform layer; ONL, outer nuclear layer. Scale bars: 50 μm.

Immunohistochemical staining of p-AMPK revealed its distribution in the GCL and the IPL (Fig. 7), and colocalization with NeuN identified p-AMPK in the cytoplasm of RGCs. The expression of p-AMPK was significantly increased in the RGCs (Fig. 7, arrowheads) and in the IPL following induction of diabetes at weeks 1, 4, and 8. Our results also showed an increase in p-AMPK expression in the RGCs (Fig. 7, arrowheads) following glaucoma induction, although this increase was less apparent compared with the diabetic retinas. The changes in p-AMPK expression in the IPL were also not observed in the diabetic retinas.

### Effect of autophagy inhibition on RGC death

**Figure 8:**
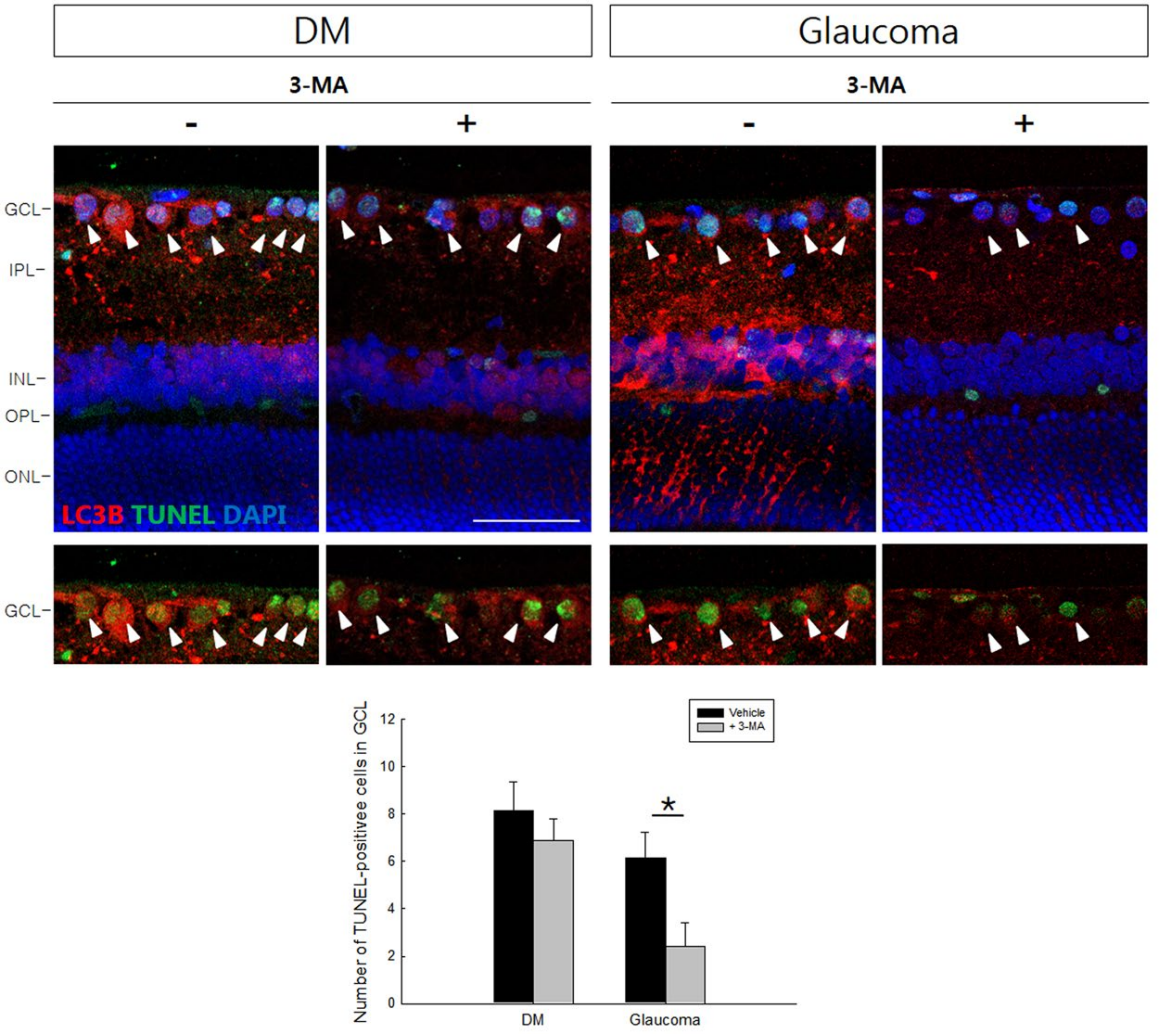
After treatment with 3-methyladenine (3-MA), LC3B expression was significantly decrease throughout the diabetic and glaucomatous retinas. TUNEL-positive cells in the GCL significantly decreased after treatment with 3-MA at 8 weeks after the induction of glaucoma, however, the effect was minimal in the diabetic retina after treated with 3-MA. Six eyes were used for each experimental period. Cells were counted at 400×magnification. Bar represents the mean ± SD. *p < 0.05 compared with the control. Scale bars: 50 μm.

We inhibited autophagy with 3-methyladenine (3-MA) and found that TUNEL-positive cells were similar before and after the inhibition of autophagy in the GCL of diabetic retinas (Fig. 8). However, the number of TUNEL-positive cells in the glaucomatous retinas significantly decreased when autophagy was inhibited.

## Discussion

Our results suggest that autophagy is activated in RGCs in both diabetic and glaucomatous retinas; however, its role in RGC survival appears to differ in two conditions. Chronic elevation of IOP in glaucoma triggers a gradual increase in beclin-1 and LC3B-II in the absence of AMPK involvement. Inhibiting autophagy rescues RGC apoptosis, which suggests that the autophagy-dependent cell death of RGCs is important in glaucomatous retinas. Induction of diabetes also initiated autophagy and resulted in an increase in LC3B-II in RGCs with AMPK activation. However, inhibiting autophagy in the diabetic retina resulted in no changes of RGC apoptosis, indicating that, in this case, autophagy may act as a survival attempt initially to restore cellular energy, however, fails.

Many studies have investigated the role of autophagy in RGC survival. The activation of autophagy following acute injury, such as optic nerve transection or crush, promotes cell survival in glaucoma^21,22^. The level of LC3B-II protein was increased following injury, and rapamycin, which induces autophagy, significantly increased RGC survival following optic nerve transection^22^. Acute injury to the optic nerve induces not only growth factor deprivation but also profound calcium release, which may trigger RGC death. In this situation, autophagy appears to promote cell survival by restoring energy levels. Following chronic, moderate elevation of IOP, LC3B-II was increased in both the short-term and long-term in rat and monkey glaucoma models^18,29^. The inhibition of autophagy with 3-MA protected RGCs, indicating that autophagy may prevent apoptosis following chronic IOP elevation^18^. The death of RGCs following chronic IOP elevation is primarily due to growth factor deprivation, which is caused by mechanical obstruction of axoplasmic flow in the RGCs. This injury downregulates PI3K pathway and decreases Bcl-2/Bcl-xL, which may release beclin-1 and induce beclin-1-related autophagy^19,20^. The interaction between beclin-1 and Bcl-2 family members, which is considered an important mechanism of stimuli-induced autophagy regulation, may be important following chronic elevation of IOP elevation. However, chronic IOP elevation induces not only growth factor deprivation but also glial cell activation in the retina. Activated glial cells are thought to secrete tumor necrosis factor (TNF), which acts as an extrinsic cell death pathway for RGCs. In a study using a glaucoma model with chronic elevation of IOP, upregulated TNF was shown to be significantly decreased following rapamycin treatment, providing RGC protection^30^. Altogether, theses studies indicate that the role of autophagy may differ depending on the triggering injury and the relevant cell death pathway.

Ischemia results in various changes in autophagy. Acute retinal ischemia–reperfusion models have shown that LC3B-II levels are significantly decreased at the end of ischemia and are maintained at below basal levels in the retina during the reperfusion^23^. After 24 h of ischemia, there was no difference in the levels of LC3B-II^23^. However, another study showed an increase in LC3B-II after 24 h of ischemia^24^. Rapamycin induction of autophagy in diabetic retinopathy, which results in chronic ischemic changes in the retina, angiogenesis, decreases vascular endothelial growth factor production, and suppresses retinal oxidative stress^31,32^. Ischemia to the retina induces glucose deprivation or oxidative stress, and AMPK plays a pivotal role in energy homeostasis and metabolism under these conditions^33^. When RGCs are under energetic stress due to ischemia, and the cytosolic availability of ATP and adenosine monophosphate (AMP) changes, activated AMPK results in activation of energy-producing pathways, including autophagy^34,35^. Our study has also shown that AMPK is activated in the diabetic retina, and inhibiting AMPK-induced autophagy has minimal effect on RGC apoptosis. Similarly, activating AMPK-induced autophagy through mTOR pathways has been shown to play a protective role in ischemic heart and cerebral diseases^36–38^. However, chronic elevation of IOP resulted in autophagic cell death without involvement of the AMPK pathway, which differed from the changes in the diabetic retina. Although further investigation will be required, we believe that different triggering injuries may activate different pathways, resulting in different roles of autophagy in RGC death.

In summary, our study demonstrated that chronic IOP elevation is associated with autophagy activation, resulting in RGC death. However, in the diabetic retina, AMPK activation induced autophagy that may serve as a survival attempt of RGCs. Thus, the triggering injury appears to regulate the role of autophagy differentially in different ocular diseases. This will be an important consideration in the development of therapeutic strategies to protect RGCs in glaucoma and diabetic mellitus.

## Methods

### Animals

We used 7–8 weeks old and 250–300 g weighted adult male Sprague–Dawley rats in this study. Experimental and control groups consisted of six animals for each time period and procedure. Three animals were used for each time period for TEM. We complied with the ARVO statement for the Use of Animals in Ophthalmic and Vision Research during the animal experiment. We considered the National Institutes of Health Guide for the Care and Use of Laboratory Animals (NIH Publications, no. 80–23, revised 1996). Additionally, the animals were care by the regulations of the Catholic Ethics Committee of the Catholic University and the Institutional Animal Care and Use of Committee of the Catholic University of Korea approved our experimental protocols. Glaucoma were induced in one group of rats and diabetes mellitus was induced in the other group of rats. Total of 116 animals was used. Animals were treated with 3-MA in each group. Intravitreal injection of 30 μg 3-MA melted in 10 μL of saline was performed. To minimize the number of animals, careful management of animals and procedures were performed.

### Glaucoma rat models

Anesthesia was performed by intraperitoneal injection of 50 mg/kg ketamine with zolazepam (Zoletil; Virbac, Carros, France) and 15 mg/kg xylazine hydrochloride (Rompun, Bayer, Leuverkeusen, Germany). Cautery of three episcleral veins were performed using a surgical microscope (Olympus, Tokyo, Japan). Then, normal perfusion of the retina was checked using planar ophthalmoscopy after cauterization. IOP was measured using a Tono-pen (Solan, Florida, USA) after topical anesthetization with Alcane (Alcon Laboratories, Fort Worth, Texas, USA). Eyes that did not present any complications during surgery or scleral burns were used.

### Diabetic rat models

Diabetes was induced by a single intraperitoneal injection of 60 mg/kg STZ (Sigma, St. Louis, MO, USA) in a citrate buffer solution (0.1 mol/L citric acid and 0.2 mol/L sodium phosphate, pH 4.5). Age-matched control rats received an equivalent volume of the citrate buffer solution. The blood glucose levels of each rat were measured using an automated Accu-Chek glucometer (Roche Diagnostics; Indianapolis, IN, USA) 3 d after STZ injection. Animals with a plasma glucose reading of >350 mg/dL were considered diabetic and used for further experimentation. Weights and blood glucose levels were recorded once per week following the induction of diabetes.

### Immunohistochemistry

Immediately after the animal scarification, eyes were enucleated and fixed in 4% paraformaldehyde at 4 °C for 10 min; the anterior segment was removed, the posterior was fixed in 4% paraformaldehyde for 60 min and pre-embedded in 3% agar. Vibratome sections (50 μm) were obtained. After several washing with phosphate-buffered saline, blocking with 10% normal donkey serum in PBS for 1 h at room temperature was performed. The slides were then incubated with rabbit anti-beclin-1 (Cell Signaling, Danvers, MA, USA), anti-LC3B (Sigma), anti-phosphorylated AMPK (p-AMPK; Cell Signaling), anti-AMPK (Cell Signaling), anti-phosphorylated mTOR (p-mTOR; Cell Signaling), and anti-mTOR (Cell Signaling) antibodies overnight at 4 °C. Then, sections were incubated with goat anti-rabbit Alexa 546 antibody (Molecular Probes, Eugene, CA, USA). For double-labeling studies, sections were further incubated with mouse anti-NeuN (Chemicon, Temecula, CA, USA) and goat anti-mouse Alexa 488 (Molecular Probes, Eugene, CA, USA). Finally, the slides were mounted with Vectashield mounting media with DAPI (Vector Laboratories, Burlingame, CA, USA). Image acquisition was performed using confocal laser scanning microscopy (Zeiss, Germany).

### Western blot analysis

Control and injured retinas were lysed in ice-cold RIPA buffer [50 mM Tris-HCl pH 7.5, 150 mM NaCl, 1 mM EDTA, 0.1% SDS, 1% IGEPAL and 0.5% sodium deoxicholate] containing protease and phosphatase inhibitor cocktails. Lysates were centrifuged for 25 min at 10,000 × g at 4 °C. Supernatants were assayed for protein content by a standard bicinchoninic acid assay (Pierce, Rockford, IL, USA). Equal amount of total proteins from the retinal extracts (40 μg) was separated by SDS-polyacrylamide gel electrophoresis and nitrocellulose membrane (Hybond-C, Amersham Pharmacia Biotech, Germany), and the blots were stained with Ponseau S (Sigma). The membranes were blocked with 5% non-dried skim milk in Tris-buffered saline with Tween buffer (20 mM Tris-HCl pH 7.6, 137 mM NaCl, and 0.1% Tween 20) for 45 min. Blots were probed for 24 h with antibodies against beclin-1 (Cell Signaling), LC3B (Sigma), p-AMPK (Cell Signaling), AMPK (Cell Signaling), p-mTOR (Cell Signaling), mTOR (Cell Signaling), and GAPDH (Sigma). The blots were then probed with goat anti-rabbit secondary antibody for 1 h at room temperature. Protein bands were visualized with the chemiluminescence system (Amersham, MA, USA) and X-ray film. Intensity of the bolts was measured using an ImageMaster VDS (Pharmacia Biotech, CA, USA), and the fold changes in protein levels compared with GAPDH were calculated.

### Transmission electron microscopy

Retinal sections were fixed by immersion in Kamovsky’s solution for 24 hr at 4 °C, processed, and embedded in acrylic resin. Ultrathin sections (0.1 μm) were prepared, mounted on Formvar-coated slot grids, and stained with 3% lead citrate. Examination was performed with Zeiss transmission electron microscope (Zeiss Inc., Thornwood, NY, USA). A double-blind point-counting method was used to quantify double-membrane vacuole-like structures in 50 micrographs (2500 μm^2^ total) in each sample.

### TUNEL staining

We assessed cell apoptosis by using *In Situ* Cell Death Detection Kit (Roche Applied Science) following the manufacturer’s instructions. Slides were mounted using Vectashield mounting media with DAPI (Vector Laboratories, Burlingame, CA, USA). Examination was performed using confocal laser scanning microscopy (Zeiss).

### Statistical analysis

Data are expressed as mean ± standard deviation (SD). Differences among groups were analyzed using Student’s *t*-test. A probability value of <0.05 was considered to represent a statistically significant difference.

## Electronic supplementary material

Supplementary Information
