# Supplementary material for: Different contributions of autophagy to retinal ganglion cell death in the diabetic and glaucomatous retinas
Source: Sci Rep. 2018 Sep 6;8:13321. doi: 10.1038/s41598-018-30165-7 (PMC6127281; doi:10.1038/s41598-018-30165-7)
Supplement: Supplementary file 1 — Supplementary Information [file 41598_2018_30165_MOESM1_ESM.pdf]

Supplementary figures for the manuscript entitled as follows

Different contributions of autophagy to retinal ganglion cell death in the diabetic and glaucomatous retinas

Hae-Young Lopilly Park, Jie Hyun Kim, Chan Kee Park

Supplementary Figures S1

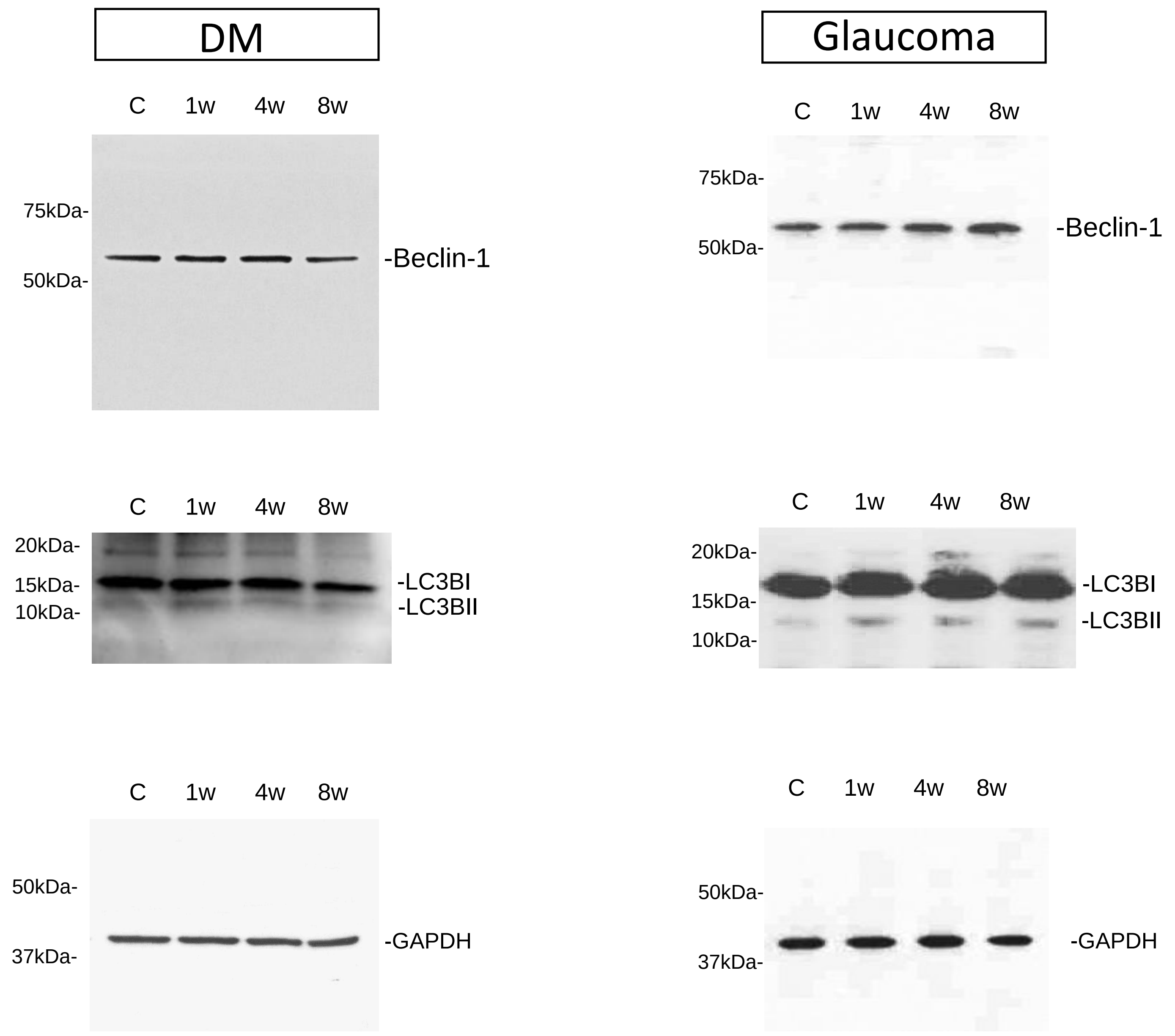

Full length blots of Figure 2

Supplementary Figures S2

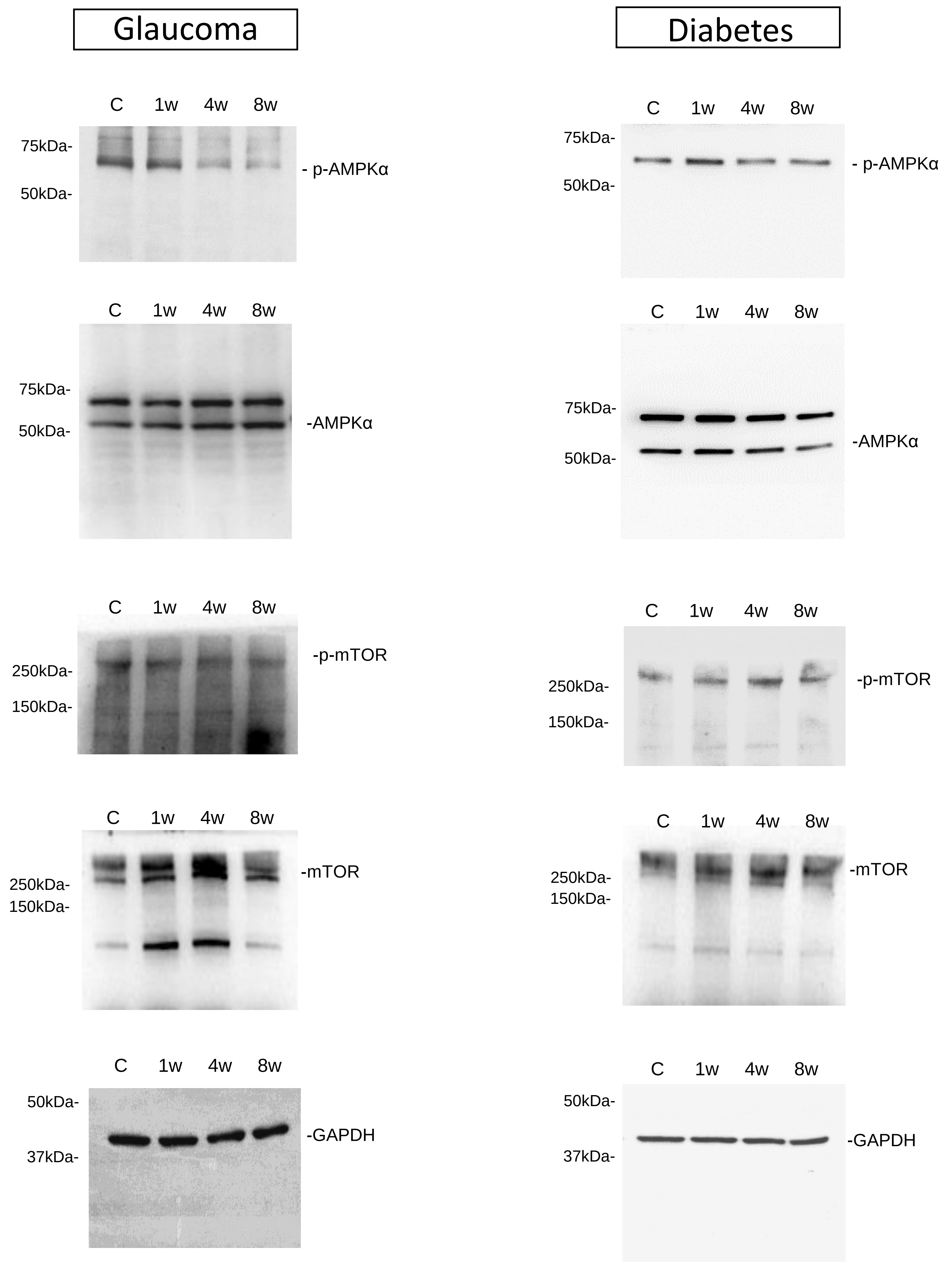

Full length blots of Figure 6
